# Biological Control beneath the Feet: A Review of Crop Protection against Insect Root Herbivores

**DOI:** 10.3390/insects7040070

**Published:** 2016-11-29

**Authors:** Alan Kergunteuil, Moe Bakhtiari, Ludovico Formenti, Zhenggao Xiao, Emmanuel Defossez, Sergio Rasmann

**Affiliations:** Functional Ecology Laboratory, Institute of Biology, University of Neuchâtel, Rue Emile-Argand 11, 2000 Neuchâtel, Switzerland; alan.kergunteuil@unine.ch (A.K.); mojtaba.bakhtiari@unine.ch (M.B.); ludovico.formenti@unine.ch (L.F.); zhenggao.xiao@unine.ch (Z.X.); emmanuel.defossez@unine.ch (E.D.)

**Keywords:** biological control, root pests, soil fauna, belowground plant defenses, tri-trophic interactions

## Abstract

Sustainable agriculture is certainly one of the most important challenges at present, considering both human population demography and evidence showing that crop productivity based on chemical control is plateauing. While the environmental and health threats of conventional agriculture are increasing, ecological research is offering promising solutions for crop protection against herbivore pests. While most research has focused on aboveground systems, several major crop pests are uniquely feeding on roots. We here aim at documenting the current and potential use of several biological control agents, including micro-organisms (viruses, bacteria, fungi, and nematodes) and invertebrates included among the macrofauna of soils (arthropods and annelids) that are used against root herbivores. In addition, we discuss the synergistic action of different bio-control agents when co-inoculated in soil and how the induction and priming of plant chemical defense could be synergized with the use of the bio-control agents described above to optimize root pest control. Finally, we highlight the gaps in the research for optimizing a more sustainable management of root pests.

## 1. Introduction

Agricultural land covers 25% of the Earth’s terrestrial surface and is one of the major drivers affecting global ecosystem health [1]. The transformation of agriculture after World War II led to the modern conventional approaches. However, the ecological costs of such agriculture have been largely underestimated, if not ignored, and evidence with respect to the actual limits of conventional agriculture regarding crop productivity continue to accumulate [2]. In the context where three billion kilograms of pesticides are annually applied worldwide and suspected to result in 220,000 deaths per year [3], numerous legislations have been implemented to reduce the use of wide-spectrum insecticides in order to protect both environmental and human health, although further efforts are required in this direction [4]. More sustainable approaches are, therefore, needed to resolve agronomic challenges while also reducing chemical pollution [5].

While insect herbivory causes severe damage to plant production in natural systems, their impact on agroecosystems is even more pronounced due to landscape simplification (e.g., loss of plant diversity and reduction of trophic interactions) [6]. Indeed, annual crop losses from damage caused by insects could be more than 15% [7]. In this context, it is worthwhile to consider below-ground herbivores that sustain a wide diversity and feed on various plant tissues, such as roots, rhizomes, and storage organs [8,9]. Root pests have always caused extensive damage to crops. For instance, the aphid root-feeding *Daktulosphaira vitifoliae*, the grape phylloxera, had almost destroyed the entire European grape production [10], and root herbivores are still responsible for a large part of yield loss at the global scale [11]. Indeed, root pests, such as wireworms (Coleoptera: Elateridae), feed on a wide range of crops, including cereals, potato, carrot, sugar beet, and fruit orchards [12]. The cost of damage caused by the western corn rootworm (*Diabrotica virgifera virgifera*) in Europe and in USA, could be much greater than $1 billion annually [13]. In the southern hemisphere, the damage caused by the greyback canegrubs (*Dermolepida albohirtum*) cost over $10 million to sugarcane producers [14]. Despite the economic importance of root herbivores, research aiming at developing sustainable solutions to diminish their impact remains scarce, compared to those pertaining to above-ground herbivores. One of the major reasons is certainly their unclear life cycle, which leads to the “out of sight, out of mind” paradigm, as argued by Hunter [8]. Indeed, their development in soils complicates the detection of infestations and, consequently, the resultant damages over the economic thresholds are generally disclosed much later than useful. In addition, even when they are readily detected, their underground mode of life limits the control of root herbivores by chemical inputs, which generally requires direct exposure to bio-active compounds. On the other hand, since the dispersion of root herbivores is comparatively limited in soils, they are more persistent locally, as compared to above-ground pests [15]; this would favor constant and localized applications of bio-control agents in the field.

**Figure 1:**
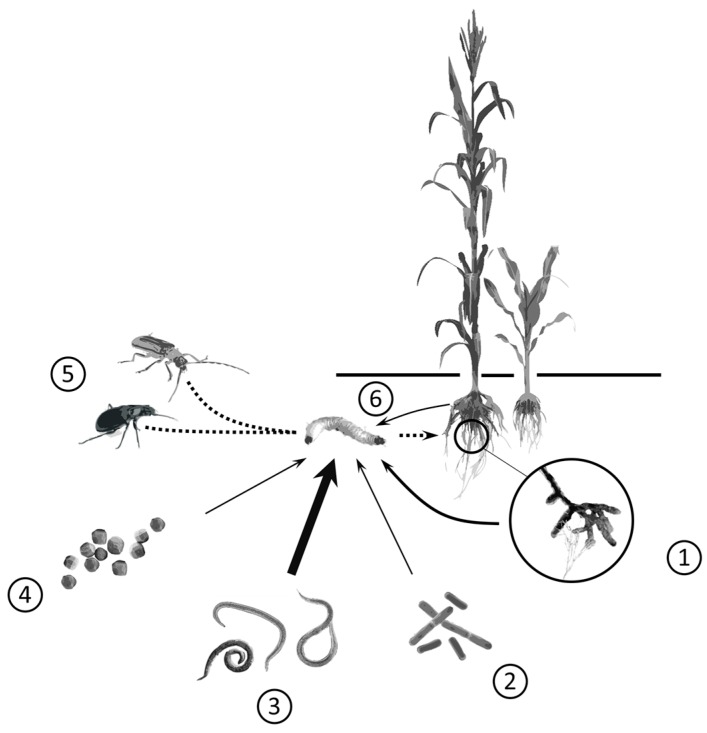
Biological control agents against root insect pests. (**1**) Entomopathogenic fungi (occasionally endophytic); (**2**) free-living soil microbes, such as *Trichoderma* or *Bacillus* spp.; (**3**) entomopathogenic nematodes; (**4**) viruses, such as *Baculovirus*; (**5**) arthropod predators; and (**6**) plant endogenous defenses and priming. Arrows represent trophic links. Different line thicknesses represent strengths of interactions, with thicker lines representing stronger potential biocontrol effectiveness than thinner lines. Dashed lines represent the currently weakest potential for biological control agents, as described in the text.

From an ecological perspective, biological pest control relies on two main forces: bottom-up (i.e., the effect of plants on herbivores) and top-down pest control (i.e., the effect of predators and parasites on herbivores) [16,17]. Since a myriad of soil organisms feed on root herbivores, they could be used as bio-control agents for root pest control in top-down control approaches. In the present review, we summarize the use of microfauna (body width <100 µm) and macrofauna (body width >2 mm) in below-ground biological control (Figure 1). Microfauna is the most important biomass in soils and represent a vast reservoir of bio-control agents. Herein, we highlight specifically the efficiency of viruses, bacteria, fungi, and nematodes in crop protection. Currently, the members of the macrofauna do not include commercially available bio-control agents, but some species could be promising in root pest control under certain conditions. In the first of seven sections, we present examples relying on either inundative (mostly microfauna) or conservation biocontrol (mostly macrofauna) when bio-control agents are not commercially produced, but are subject to considerable efforts to enhance their activity within crops. In the eighth section, we then discuss how pest control could be increased via the induction of plant defense mediated by elicitors or soil microfauna (bottom-up effect). Finally, we discuss the main perspectives relevant to the promotion and improvement of below-ground biocontrol in the near and distant future.

## 2. Viruses

It has been estimated that the “virosphere” of the Earth’s oceans encompasses over 10^30^ viruses, and soils, by virtue of their diversified habitat system, could shelter even larger populations [18]. Viruses can practically infect the entire gamut of living organisms, and ecologists have long been interested in understanding their role in regulating insect populations. Three decades ago over 650 entomopathogenic viruses were already isolated from insects [19].

Currently, entomopathogenic viruses belonging to the family baculovirus, a family of dsDNA viruses, are the main group of arthropod viral pathogens. They have been isolated from 700 species of arthropods and include the most promising viruses for insect biological control [20,21,22,23,24]. Thus far, baculoviruses have been included in about 60 commercially available products [25]. Baculoviruses produce characteristic occlusion bodies ensuring better virus survival in the environment and, more importantly, enabling good insect infestation [26]. After ingestion by insects, occlusion bodies are dissolved in the alkaline midgut, and the released virions initiate the infestation through epithelial cells before contaminating the entire organism. Soils represent the most important reservoirs of occlusion bodies and are crucial environmental compartments involved in the control of insects completing a part of their life cycle under the soil surface [27]. The high diversity within baculovirus results from a long coevolution with insects and has led to narrow host specificity [28]. As a consequence, they exert limited adverse effects on non-targeted species. Despite successful bio-control programs towards above-ground pests, viral insecticides targeting root pests are rare, and applied research exploring the potential of virus for below-ground biocontrol remains scarce.

To our knowledge, only one example illustrates the efficiency of baculoviruses against insect root herbivores: the use of the potato tuberworm granulovirus (PoGV, family Baculovirus, genus *Granulovirus*) to control the potato tuberworm complex (Table 1). The agronomical interest of PoGV against *Phthorimaea operculella* (Lepidopetra; Gelechiidae) has been validated by governmental agencies in different countries of South America and tested in North Africa, Asia, and the Middle-East [29]. *Phthorimaea operculella* is a worldwide pest of solanaceous crops, which can cause up to 100% economic losses, since potato tubers containing larvae are generally considered unmarketable. During the growing season, females preferentially lay eggs on leaves or on tubers when available. Larvae mine leaves and dig galleries throughout the stem before reaching the tubers where they continue to develop even after harvest [30]. Based on the life cycle of *P. operculella*, the biocontrol of potato tuberworm mediated by PoGV can be achieved either in crops or in post-harvested potato tuber stores. Successful controls have been established for both strategies since spraying of PoGV at the soil surface reduces 73% of tubercle infestation in crops [29,31], while formulations of PoGV applied on stored tubers led to between 53% and 100% *P. operculella* mortality [30,32]. A second pest species belonging to the potato-tuberworm complex is *Tecia solanivora* (Lepidopetra; Gelechiidae), which recently invaded the northern part of South America [33]. Interestingly, while different isolates of PoGV have been selected for their infectivity towards either *P. operculella* or *T. solanivora*, it has been shown that the combination of these isolates increases the control efficiency of both tuberworm species compared to a single application of these isolates [34].

In order to develop marketable viral-insecticides, it would be necessary to overcome several challenges. For instance, insect-specific resistances continuously evolve, and important variations in host infectivity have been recorded between different strains of PoGV [35]. Consequently, companies producing viral bio-insecticides should pay attention to select strains of viral agents that remain highly infectious toward pests. Indeed, the recent emergence of baculovirus resistance in *Cydia pomonela* highlights the need to develop good bio-control practices for reducing the risks of pest resistance [36,37]. The success and the durability of pest bio-control rely on the selective pressures exerted by viruses on root pests and, ultimately, on the ability of those pests to develop immune systems conferring adaptations towards the biocontrol agents. In this context, biological control strategies should ideally promote the application of a mixture of viral strains harboring different mode of actions in order to diversify selective pressures and avoid (or at least delay) the development of resistances in root pests. In addition, one of the major drawbacks for the commercialization of viral bio-insecticide is the need to optimize massive production. Most of the previous programs relied on the costly approach of in vivo production. Nonetheless, in the case of the potato tuberworm biocontrol, new insights in the establishment of cell lines of *P. operculella* on artificial medium could be of great importance in developing strategies for the massive production of PoGV [38]. These technical outbreaks are required to commercialize viral insecticides with reasonable costs, as compared to chemical insecticides. Finally, different authors have stressed the importance of improving application methods. Apart from studies focusing on the appropriate density of viruses to release [32] or the optimal weather conditions for inoculating soils [18], additional efforts are required to develop efficient formulations ensuring field stability of viral insecticides. Indeed, virions of baculoviruses contained in occlusion bodies are very susceptible to ultraviolet light and sun protection additives, such as uric acid, lignin, or corn flour, have been shown to increase viral infectivity when included in the final formulation [39,40].

Recently, virologists have also been interested in increasing the effectiveness of viral bio-control agents through genetic engineering, even if none of these recombinant baculoviruses have been registered yet [20]. More particularly, it would be possible to create recombinant baculoviruses with genes encoding for scorpions’ neurotoxins in order to reduce the lethal time of pathogenic viruses [39]. However, with regard to the production costs, the interest on such hybrid bio-control agents could be limited since baculoviruses already harbor relatively rapid virulence activity by killing their hosts in 5–14 days, depending on strain specificities and environmental factors [20]. More importantly, viral strains based on genetic modifications present three main ecological limits, which are still debated in the literature. First, at the population level, further research is required to study how the balance between both natural and recombinant viruses evolves in soils in order to assess the advantage of releasing recombinant viruses on crops. Second, the co-evolution between viruses and their respective hosts trigger dynamic patterns in virus infectivity and, consequently, genetic engineering cannot be considered as a silver bullet since insect resistances are expected to be selected over the mid- or long-term. Further research is required to study the extent to which insect resistances towards recombinant viruses appear in natural populations of root pests. Moreover, hybrid viruses could lead to dramatic unknown effects at the community level since microbial communities are characterized by horizontal transmission of genes, even if such transfers have never be proved in bio-control programs [25]. In this context, the ecological impacts of genetically-modified viruses in soils need to be estimated before any large application.

## 3. Bacteria

Bacteria are ubiquitous to the environment and have evolved intimate interactions, from mutualistic to pathogenic, with a large number of studied insects [68]. Entomopathogenic bacteria are well known for their ability to produce a plethora of protein insecticidal toxins [69]. Since their discovery during the 19th century, bacterial toxins acting as virulence factors have been shown to range from very specific to broad insecticidal spectrum. In comparison with chemical insecticides, bacterial toxins show high diversity of simultaneous action, contributing to the sustainability of bacteria-based bio-pesticides by limiting insect resistances. Hereafter, we mainly discuss the use of *Bacillus thuringiensis* (Bt) representing approximately 95% of microorganisms used in biocontrol [70].

The economic success of *B. thuringiensis* is sustained by the large amount of information on its main insecticidal toxins; these are the protein-based δ-endotoxins named “Cry”, which are lethal for several species of various insect orders [71]. To date, about 170 different “Cry” toxins have been isolated, which are effective against several coleoptera, lepidoptera, and diptera species [72]. These proteins are produced upon sporulation, and are contained in crystal inclusions. Once ingested, crystals inclusions are solubilized by the insect proteases in the midgut, inadvertently activating the “Cry” proteins [73]. Interdisciplinary investigations have largely extended the array of Bt-based insecticides, from wettable powder or liquid formulation to transgenic crops, thereby facilitating their use in organic farming and integrated pest management (IPM) programs.

Most solutions based on Bt insecticides contain both δ-endotoxin crystals and spores of *Bacillus thuringiensis*. This mixture-based formulation is known to synergize the toxicity of the commercial products. Although the first commercialized Bt-insecticide, “Sporeine”, was developed in the late 1930s, this product was mainly used against an above-ground herbivore: the European corn borer, *Ostrinia nubilalis* (Lepidoptera: Crambidae). Thus far, most Bt-insecticides are derived from a single subspecies, *B. thuringiensis* subsp. *kurstaki*, which is particularly efficient towards lepidopteran pests. Bt insecticides targeting non-lepidopteran insects are far less common despite active subspecies against various insect orders, including soil-dwelling pests (Table 1). For instance, *B. thuringiensis* subsp. *israelensis* can reduce the survival of fungus gnats (Diptera: Sciaridae), an important root pest in greenhouses, to one-tenth the original and is currently commercialized to control sciaride larvae (e.g., Gnatrol^®^, Abbott Laboratories, Chicago, IL, USA; Solbac, Andermatt Biocontrol, Grossdietwil, Switzerland) [45,46]. A field study has shown that applications of the same subspecies led to 74%–83% of control of early instar of crane flies, *Tipula paludos* (Diptera: Nematocera), thereby providing interesting solutions to protect pastures and turfs [42]. In addition, a Bt insecticide based on *B. thuringiensis* subsp. *tenebrionis* (Novodor^®^, De Sangosse, Pont du Casse, France) can be used to control coleopteran larvae, such as *Epitrix tuberis* (Coleoptera; Chrysomelidae), feeding on potato tuber and, to a lesser extent, *Diaprepes abbreviatus* (Coleoptera; Curculionidae), attacking citrus roots [47,48]. White grubs represent another major root pest and experimental studies have highlighted the potential of two subspecies of *B. thuringiensis*, subsp. *japonensis* and subsp. *galleriae*, against different scarab larvae, such as *Anomala cuprea*, *Anomala orientalis* and *Popillia japonica* [43,44].

Recent advances in proteomic and molecular biology have opened new perspectives in Bt-based biocontrol against major root herbivores, such as the western corn rootworm *Diabrotica virgifera virgifera* (Coleoptera; Chrysomelidae). Indeed, technical breakthroughs have permitted the fine identification of the three-dimensional structure of the largest family of “Cry” proteins. These protein toxins are formed by three main amino acid domains involved either in cell lysis (domain I) or host specificity (domains II and III) [74]. Recently, a study has shown promising results by recombining amino acid sequences of “Cry” toxins. While the authors conserved the protein structure required for insect cell lysis, they exchanged regions in a specific domain and, consequently, developed a hybrid toxin with a new insect specificity [75]. This engineered toxin, “eCry3.1Ab”, induces over 90% of larval mortality of the corn rootworm. However, after the registration of Bt-corn lines producing “Cry” toxins, populations of *Diabrotica virgifera virgifera* have rapidly developed cross-resistances towards different “Cry” toxins, including “eCry3.1Ab” [76]. This rapid appearance of resistances may be attributed to continuous expositions over spatial and time scales. In addition, some studies have shown that genetically-modified corn plants could be responsible for the persistence of “Cry” proteins in the environment [77,78,79]. In this context, further questions related to beneficial and/or hazardous impacts on targeted and non-targeted insects remain to be addressed with caution.

While the market of bacterial-based bio-control agents is largely dominated by a single species, *B. thuringiensis*, both farmers and industries should benefit from expanding into other species [80]. For instance, *Paenibacillus popilliae*, responsible for the milky disease of white grubs has recently become commercially available to control the Japanese beetle *Popillia japonica*. In addition, *Brevibacillus laterosporus* was reported to be active against different root pests and various other plant pathogens such as mollusks, nematodes, bacteria, and fungi [80]. A generalist bio-control agent, such as this one, could be of high interest to farmers. The entire genome of *B. laterosporus* has been recently sequenced; therefore, future efforts focusing on these toxins could bring novel insights in bacteria-mediated biocontrol.

## 4. Fungi

As for the other systems, the studies exploring the potential of entomopathogenic fungi (EPF) in sustainable agriculture indicate a striking asymmetry between above- and below-ground target pests. Thus far, EPF have been mainly investigated for their role in controlling above-ground pests. Except for a few pioneering studies showing, for instance, that Beauveria bassiana can efficiently infect root weevil (Diaprepes abbreviatus) larvae [56], EPF have only recently been considered for controlling root-feeding pests [51]. As shown in Table 1, the most common commercially available EPF-based products to control root pests include three genera of opportunistic insect pathogens: Beauveria (Hypocreales: Cordycipitaceae) with products such as Naturalis® (Intrachem Bio Italia, Grassobbio, Italy) (Beauveria bassiana ATCC 74040 isolate), Metharizium (Hypocreales: Clavicipitaceae) with products such as Met52® (Novozymes, BagsvaerdDenmark) and BioCane™ (Bio-Care Technology, Somersby, Australia) (Metharizium anisopliae), and Isaria (Hypocreales: Cordycipitaceae) with products such as PreFeR-al® WG 8 (Biobest, Westerlo, Belgium) (Isaria fumosorosea).

There are multiple advantages of adopting EPFs as root pest bio-control agent. First, EPF infection can occur by cuticle penetration, thereby already initiating the infestation from outside of the insects’ midgut [81]. Second, from industrial perspectives, EPFs are relatively easy to isolate from the field and to massively produce on artificial media, especially for the hyphomycetes, including *Metharizium* spp. and *Beauvaria* spp. [82]. Third, in comparison to chemical pesticides, the multiple mode of action of EPF lessens the possibility of resistance development in insects [83]. Fourth, EPF pathogenicity is specific to insects, avoiding unexpected deleterious effects on non-target plant-beneficial organisms [84,85]. In this context, the great diversity of EPF strains allows selecting the most pathogenic ones, depending on the type of root pest and environmental factors [86]. Different studies indicate that the field abiotic environment is a stronger operator of EPF strains’ pathogenicity than the intrinsic EPF pathogenicity determined in vitro. Thus, one of the problems in employing massively produced commercial EPFs can be their variation in pathogenicity when used in different climatic conditions [87]. Further, Esther et al. [88] showed that different *Isaria fumosorosea* EPF strains express different thermal tolerance towards the growth rate according to the temperature range of their geographical origins. Therefore, specific selection and commercialization of different EPF isolates adapted to different climatic conditions and soil properties can compensate for the EPFs’ potential lack of efficiency as root pest bio-control.

Alongside other bio-control agents, such as nematodes, EPFs can persist in soils over long time periods, thus ensuring a more durable effect. For instance, Pilz et al. [89] demonstrated that *Metharizium anisopliae* lasted in the soil for at least 15 months. Although the soil density of EPFs generally decreases with time [90], these bio-control agents remain viable in soil even at low quantity. *Metharizium anisopliae* can conserve up to 10% of the initial conidia application after three years in soil, and potentially increase in density reaching initial level post-inundation after infection and spread from insect cadavers [91]. Kirchmair et al. [49] also monitored the variation in EPF density after soil inoculation with *M. anisopliae* to control grapevine phylloxera. One year after soil inoculation, EPF density peaked, thereby ensuring a successful biocontrol of root pests, but bio-control agents then decreased and no further effect was recorded after three years. Finally, regarding the potential of *Beauvaria brongniartii* to control *Melolontha melolontha*, a long-term survey of EPF density has shown that bio-control agents can generally be isolated four years after the last inoculation, although EPF persistence has also been exceptionally reported after 15 years [92].

In addition to their insect pathogenic properties, some EPF species (*Metarhizum* sp., *Beauveria.* sp.) have evolved to behave as root endophytes (*Metarhizum* sp., *Beauveria.* sp.) [93,94]. For instance, saprophytic EPFs (*B. bassiana*, *M. anisopliae*) can establish colonies in plant roots even in the absence of insect hosts [95]. This colonization allows a direct transfer of nutrients such as nitrogen from an insect cadaver to the plant [96]. The incorporation of such EPF strains in agricultural practices may be incredibly promising, providing multiple simultaneous benefits, ranging from plant root defence to plant growth-promoting properties [97,98].

## 5. Nematodes

Among the most promising bio-control agents of root pests are the soil-borne nematodes that are obligate parasites of arthropods, also known as entomopathogenic nematodes (EPNs) in the families Steinernematidae and Heterorhabditidae e.g., [99,100,101]. Several species of EPN are currently used as classical, conservational, and augmentative biological control agents (Table 1). The vast majority of applied research, nonetheless, has focused on their potential as inundatively applied augmentative biological control agents [102].

The life cycle of EPN is characterized by an egg stage, four juvenile stages, and an adult stage. Only the third juvenile stage is the “infective juvenile” that is free-living in the soil, capable of surviving for several weeks in the soil, before infecting a new host individual [103]. Therefore, the only stage used in biological control is the third instar infective juvenile. EPNs can be considered good candidates for commercialization as biological control agents for several reasons: (1) they have a broad pest–insect host range; (2) they can rapidly kill the insect host; (3) they have active searching behavior using olfactory cues; (4) they can be mass produced, both in vivo and in vitro; (5) they have potential for application in integrated pest management programs; and (6) EPNs are generally considered safe for vertebrates and most non-target invertebrates, therefore minimizing the registration requirements [86,104].

In addition, EPNs could be implemented in crop production research. It was found that herbivore-damaged roots of several plants species release chemical signals in the soil that EPNs can exploit to more easily locate their insect hosts [105,106,107]. Considerable variation, however, exists in the manner in which these chemically-mediated belowground tri-trophic interactions unfold. For instance, it was found that most of the American varieties of corn have lost the ability to produce the chemical signal (the sesquiterpene (*E*)-β-caryophyllene) and the subsequent EPN attraction, whereas the European varieties retained it [108]. By genetically restoring the signal, it was possible to increase EPN attraction and increase plant protection against corn rootworm (*Diabrotica virgifera virgifera*) larvae in field trials [109]. Engineering new crops, taking into account EPN’s recruitment, might be a promising venue to explore [110,111]. However, overexpression of (*E*)-β-caryophyllene in genetically-modified corn lines has also been shown to trigger both physiological and ecological costs [112]. Indeed, from a community ecology perspective, this signal is involved in public channels of communication and can be used by different herbivores for their own benefits. Consequently, to tap the potential of such engineered plants, it is necessary to study their agronomic interests in multi-trophic contexts. Nonetheless, it might also be possible to select EPN lines that are more efficient in following belowground chemical signals [113].

While several positive attributes make EPN application promising, additional research is necessary to accelerate their use as bio-control agents. EPNs are very sensitive to abiotic constraints, such as low humidity, high UV radiation, high soil salinity, and high or low pH. In addition, EPNs are also quite sensitive to several pesticides (nematicides, fumigants, and others) [104]. Therefore, several factors linked to formulation, shelf life, and application optimization still inflate the overall costs of production when compared to those of chemical pesticides [101], but several promising venues are underway. For instance, a prospect of applying EPNs in the field is to explore the possibility of formulating them into capsules made from bio-compatible and bio-degradable natural polymers [114,115,116]. This should provide EPNs with a physical protection against abiotic and biotic (i.e., their natural enemies such as fungi and bacteria) sources. In addition, the efficacy of EPNs for the biological control of root pests may be enhanced by co-encapsulation of EPNs with other ingredients that may divert insect feeding from the roots of crop plants towards eating EPN-based capsules [117].

## 6. Macrofauna

In addition to the inundative strategies of biocontrol mentioned above, conservation biological control tactics for preserving soil macro-fauna has also been reported as a key component of sustainable biological control strategies [118,119,120]. Indeed, soil food-webs include a wide array of—mainly generalist—predators of herbivore pests, including carabid, centipedes, mites, spiders, and beetles [119]. For example, soil surface-dwelling ground beetles (Carabidae) and wolf spiders (Lycosidae) have been shown to depress populations of Cicadellidae and Thysanoptera in cornfields [121], while the laelapid mite (*Cosmolaelaps simplex*) requires feeding on root pests such as *Caloglyphus rodriguezi* to successfully reproduce [122].

To date, however, only a relatively small number of commercial products based on arthropod predators have had success (Table 1). Several reasons have been advanced for this, some of which are as follows: interactions between predators and their prey are difficult to predict when considered within multi-trophic systems that are under the influence of constantly changing biotic and abiotic parameters. Basically, soil environment, predator species, rate of development, density and host plant all have a considerable effect on the establishment and activity of biological control agents for root herbivores [123]. Therefore, it is not surprising that biological control using generalist predators, which are influenced by the plethora of abiotic and biotic factors, may have been limited [118]. In this context, Lee and Edwards [65] showed that in laboratory conditions, five different carabidae species can consume various immature stages of the black vine weevil, *Otiorhynchus sulcatus* (Coleoptera: Curculionidae) occurring at the soil surface, although they were not efficient in controlling the root pest in the field; this is likely due to the burring behavior of pest larvae. Moreover, in some cases, arthropod predators even produced positive effects on target pests. For example, the generalist predatory mites *Gaeolaelaps aculeifer* increased the density of corn rootworm larvae and induced higher root damage in maize [67].

In order to improve and develop commercial products based on the soil macro-fauna, several venues could be investigated. For instance, field assays integrating various ecological parameters could help identify the role of trophic linkages within belowground communities. Second, it might be important to elucidate the role of these predominantly generalist natural enemies in order to improve their efficiency. Indeed, generalist predators may attack not only targeted herbivores, but also the herbivores’ specialist natural enemies. Finally, using diverse predator communities rather than targeting conservation efforts at specific key predator taxa and employing integration methods with other bio-control agents could promote the efficiency of controlling root herbivore pests within subterranean systems [66].

## 7. Synergies between Different Bio-Control Agents

The combinations of different organisms that can synergistically work together to protect plant from root pest seems a promising way to undertake for successful belowground pest control [50,124,125]. For instance, Tinzaara et al. [126] showed that EPFs, combined with the aggregation pheromone of the banana root borer (*Cosmopolites sordidus*), improved *Bauveria bassiana* dissemination in the field and increased root pest infection by the fungus. Similarly, the combination of EPNs and other control agents has proved to be synergistic and produces higher mortality than the individual agents. For example, Koppenhofer and Kaya [127] showed additive and synergistic interactions between EPNs and *Bacillus thuringiensis* for scarab grub control. Several studies have also highlighted synergisms between EPNs and the neonicotinoid insecticide imidacloprid [128,129,130]. On the contrary, Cappaert and Koppenhofer [131] observed antagonism between imidacloprid and the EPN *Steinernema scarabaei* for controlling the European chafer (*Rhizotrogus majalis*).

Along the same lines, the simultaneous use of generalist macrofaunal predators, in addition to microbial bio-control agents, can promote the control efficacy against root herbivores. For instance, in mesocosm studies, the control of soil-dwelling stages of the western flower thrips (*Frankliniella occidentalis*) was significantly improved when predator rove beetle (*Dalotia coriaria*) and *entomopathogenic fungi* (*Metharizium anisophilae*, Met52) were combined, thereby achieving >90% thrips mortality [132].

Further macro soil fauna organisms, such as earthworms, can provide a major source of alternative food for polyphagous predators, such as carabid beetles. Indeed, earthworms have been shown to provide an ideal alternative prey for *Pterostichus melanarius* beetles when pest numbers are too low, and set them ready to switch back to feeding on arthropod pests when they become available [119]. Additionally, it was suggested that earthworms might function as a vector of insect pathogenic fungi [91] as well as dispersal agents of baculovirus occlusion bodies in the soil [27]. Therefore, earthworms not only enhance soil nutrient composition and subsequent plant growth [133], but could also indirectly facilitate pest control of root pest by natural enemies.

## 8. Interactions between Belowground Top-down and Bottom-up Forces

As discussed above, plants can recruit natural enemies of the insect’s herbivores for their own benefit (top-down control). In addition, plants can directly reduce herbivore impact through the expression of defenses, including mechanical barriers and toxic chemicals (bottom-up control) [7]. While some of these direct defenses are constitutively expressed, most direct defense traits are increased, or even de novo induced, only after herbivore attack [134]. Specifically, root responses to herbivory are controlled by the activation of a highly complex phytohormonal signaling network that includes jasmonic acid (JA), salicylic acid (SA), ethylene (ET), and abscisic acid (ABA) pathways, among others e.g., [135,136,137,138]. In the context of pest control, the manipulation of inducible resistance traits that become activated upon attack offers promising perspectives [139,140]. As for shoots, the JA pathways can also be induced in roots following root-feeder attacks although to a far lower extent [138,141]. However, higher sensitivity to this hormone and/or alternative signals in below-ground organs could compensate the reduced burst in JA after root herbivory [142]. In this context, it has been advocated that inducing (or “priming”) the seeds with chemicals, such as JA, SA, or β-amino butyric acid (BABA), can increase plant resistance against both biotic and abiotic stress [143,144,145,146].

Although such strategies have been developed mainly against pathogens, e.g., [139,147], there have been a few studies that have shown the potential of plant-induced defense against root pests. For instance, it has been shown that root herbivore attack induces jasmonate signaling in rice crop roots, and exogenous jasmonate application to the roots could enhance rice resistance against root pests [148]. A recent study by Erb et al. [149] revealed that the corn rootworm *Diabrotica virgifera virgifera* strongly avoided leaf-infested plants by *Spodoptera littoralis*. The avoidance was determined to be by recognizing systemic changes in soluble free and soluble conjugated phenolic acids. From an applied point of view, these findings show promising potential to improve the management of the corn rootworm in two ways. First, alteration of the root phenylpropanoid biosynthesis may trick *D. virgifera virgifera* into feeding on low quality (leaf-infested) host plants, which may reduce its performance and overall damage in the field. Second, there might be a possibility of mimic leaf infestation, which may deter western corn rootworm from feeding on corn roots.

Alongside a “priming strategy” based on synthetic elicitors, interactions between plant and beneficial microfauna could limit the development of root pests by inducing phytohormonal defense pathways, including JA, SA, ET, and other metabolites [150]. Such induction is often divided into two main categories: systemic acquired resistance (SAR) and induced systemic resistance (ISR). SAR is mediated by a SA-dependent process and can be induced by treatment with a variety of agents or certain chemicals (e.g., acibenzolar-S-methyl, ASM). ISR, on the other hand, is mediated by JA- and ET-sensitive pathways [151] and can be induced in plants by the application of a variety of abiotic or biotic agents, such as certain strains of plant growth-promoting rhizobacteria (PGPR) as well as non-pathogenic rhizobacteria [152,153]. Resistance-inducing and antagonistic rhizobacteria could be good candidates for formulating new inoculants, for biological control of plant disease [153]. Apart from bacteria, arbuscular mycorrhizal fungi (AMF) are another group of microorganisms that can affect root-feeding insects via indirect plant-mediated effects on the defense chemistry of plants [107,154,155]. Firstly, root colonization by AMF appears to promote direct plant defenses (such as induced secondary defensive metabolites) against herbivores [156]. For example, the production of root volatiles and, in particular, the volatile products resulting from glucosinolate or cyanogenic glycoside conversion, i.e., cyanides and isothiocyanates, have been found to be toxic or noxious to a wide range of belowground herbivores and pathogens e.g., [157,158,159]. Secondly, the volatiles produced by plants in combination with AMF can promote indirect plant defenses (i.e., the attraction of natural enemies of the herbivore) [160,161,162,163,164,165,166]. To our knowledge, these strategies, although environmentally sound and promising, are at the very early stages of implementation, and future research should focus on integrating plant-herbivore-microbe interactions into sustainable agricultural practices.

Despite the interesting synergisms between bottom-up and top-down forces regulating root herbivore populations, it is important to note that antagonistic interactions can also occur depending on specific properties of tri-trophic organisms. Although secondary metabolites involved in direct plant defenses are generally expected to be detrimental towards pests, some herbivores, mainly specialized pests, can sequester these toxic compounds to defend themselves against their natural enemies [167,168]. Hence, the ability of herbivores to redirect plant defenses against biological control agents should be taken into account when setting up bio-control strategies. For instance, it has been shown that the ability of spotted cucumber beetles to store plant defensive terpenes in their eggs limit EPF pathogenicity [169]. In consequence, biological control programs based on several bio-control agents should diversify the selective pressures exerted on herbivores and, consequently, attenuate specific drivers leading to the accumulation of toxins by pests, especially when natural enemies vary in their susceptibilities to those compounds.

## 9. Conclusions

While the benefits and costs of biological control are often expressed relative to chemical insecticides, it has been estimated that the former present a much better success ratio coupled with a far lower developmental cost [170]. Additionally, pest resistances to bio-control agents have been rarely described, thus offering appropriate sustainable solutions to control herbivore populations in the field [171]; however, some authors, such as Bardin and colleagues, have raised some concerns in this regard [172]. Nevertheless, insecticide markets remain largely dominated by chemical compounds. For instance, microbial bio-control agents including viruses, bacteria, and fungi, represent only 2% of the total insecticide market [39]. This low proportion mostly relies on their highly specific spectrum, thereby limiting their widespread use in pest control strategies, unlike chemical controls. Nonetheless, the same characteristics also confer environmentally-friendly properties by reducing adverse effects on non-targeted organisms.

From ecological perspectives, a surge in research aimed at defining the roles of soil-beneficial organisms in nature could expand the range of potential bio-control agents against root pests. Although microbial agents are mainly restricted to a few taxa (baculoviruses, *Bacillus thuringiensis* and Hypocreales for viruses, bacteria, and entomopathogenic fungi), we have reported some promising additional bio-control agents. Bacteria (e.g., *Pseudomonas* spp.) and fungi, displaying both entomopathogenic and plant mutualistic properties, may benefit crops by providing multiple services, including plant defense priming and the resulting bottom-up pest control. Currently, nematodes are certainly the most widely adopted bio-control agents against root pests and they have been used in several successful programs. On the contrary, the biological control of root herbivores based on macro-fauna has yielded unsatisfactory results so far. We argue that conservation efforts of generalist predators, such as ground beetles, may focus on ecological niches of guilds rather than on single species, while the combination of strategies including microbial agents should be advocated. 

The crosstalk between academic and industrial sectors is imperative to improve root pest control. For instance, applied research should pay more attention to the timing of applications in order to maximize the activity and the stability of bio-control agents in the environment, especially when weather conditions can dramatically affect the efficiency of the microbial agents [18]. In addition, recent insights on the encapsulation of microbial agents should rapidly lead to innovative solutions when applying nematodes, bacteria, fungi, or viruses [115]. From the industrial perspective, massive production of bio-control agents is certainly one of the major limitations. Further research aimed at establishing bio-reactors may help develop strategies to overcome this drawback [173].

Finally, we advocate the application of a combination of approaches for effectively reducing root pest populations. In this context, integrated pest management spanning soil biodiversity and health conservation, in conjunction with innovative application of bio-control agents, should offer an appropriate framework to efficiently control root pests.

## Figures and Tables

**Table 1:** Biological control agents that are currently used for crop protection against root insect pests.

Biocontrol Agents	Root-Pest Common Name	Root-Pest Scientific Name ^1^	Key Crops Targeted	Entomopathogenic Species Used ^2^	Biocontrol Method	Status	Potential Future Use	References
**Virus**								
	Potato tuber moth	*Phthorimaea operculella* (1)	Potato	Granulovirus (PhopGV)	Inundative	Government agencies	Yes	[32]
								[29]
								[33]
	Potato tuber moth	*Tecia solanivora* (1)	Potato	Granulovirus (PhopGV)	Inundative	Government agencies	Yes	[33,34]
**Bacteria**								
	Japanese beetle	*Popillia japonica* (2)	Turf	*Paenibacillus popilliae*	Inundative	Registered	Yes	[41]
	Crane fly	*Tipula paludos* (3)	Pasture, turf	Bt subsp. *israelensis*	Inundative	Experimental	Yes	[42]
	Cupreous chafer	*Anomala cuprea* (2)	Peanut	Bt subsp. *galleriae*	Inundative	Experimental	Yes	[43]
	Oriental beetle	*Anomala orientalis* (2)	Turf	Bt subsp. *japonensis*	Inundative	Experimental	Yes	[44]
	Japanese beetle	*Popillia japonica* (2)	Turf	Bt subsp. *japonensis*	Inundative	Experimental	Yes	[44]
	Fungus gnat	*Bradysia spp.* (4)	Horticulture	Bt subsp. *israelensis*	Inundative	Registered	Yes	[45]
								[46]
	Tuber flea beetle	*Epitrix tuberis* (5)	Potato	Bt subsp. *tenebrionis*	Inundative	Registered	No	[47]
	Root weevil	*Diaprepes abbreviatus* (6)	Citrus	Bt subsp. *tenebrionis*	Inundative	Registered	No	[48]
**Fungi**								
	Grapevine phylloxera	*Daktulosphaira vitifoliae* (7)	Vineyard	Ma	Inundative	Registered	Yes	[49]
	Black vine weevil	*Otiorhynchus sulcatus* (6)	Berries	Ma, Bb	Inundative	Registered	Yes	[50]
								[51]
	White grub	*Cyclocephala signaticollis* (2)	Crops, fruit, ornamentals, turf and pasture	Bb	Inundative	Experimental	Yes	[52]
	Cabbage root fly	*Delia radicum* (8)	Cabbage	Ma	Inundative	Experimental	Yes	[53]
	Banana root borer	*Cosmopolites sordidus* (6)	Banana	Bb, Ma	Inundative	Experimental	No	[54]
	Diaprepes root weevil	*Diaprepes abbreviatus* (6)	Citrus, sugar cane	If, Bb	Inundative	Experimental	No, Yes	[55]
								[56]
	Black cutworm	*Agrotis ipsilon* (9)	Turf, vegetables	Ma, Bb	Inundative	Experimental	Yes	[57]
	Greyback cane beetle	*Dermolepida albohirtum* (2)	Sugar cane	Ma	Inundative	Registered	No	[58]
	Wireworms	Coleoptera: Elateriadae	Potatoes, vegetables	Mb	Inundative	Experimental	Yes	[59]
	Onion maggot	*Delia antiqua* (8)	Bulbous plants	Ma	Inundative	Experimental	Yes	[53]
	Crane fly	*Tipula paludosa* (3)	Diff. crops	Mr	Inundative	Experimental	Yes	[59]
	Rootworm	*Diabrotica virgifera virgifera* (5)	Corn	Ma, Bb	Inundative	Experimental	Yes	[60]
								[61]
	Mole crickets	Orthoptera: Gryllotalpidae	Turf, vegetables, tree seedlings	Ma	Inundative	Experimental	Yes	[62]
	Root weevil	*Otiorhynchus* spp. (6)	Diff. crops	Bb	Inundative	Registered	Yes	[63]
**Nematodes**								
	Banana root borer	*Cosmopolites sordidus* (6)	Banana	Sc, Sf, Sg	Inundative	Registered	Yes	*
	Billbug	*Sphenophorusspp.* (6)	Turf	Hb, Sc	Inundative	Registered	Yes	*
	Black cutworm	*Agrotis ipsilon* (9)	Turf, vegetables	Sc	Inundative	Registered	Yes	*
	Black vine weevil	*Otiorhynchus sulcatus* (6)	Berries, ornamentals	Hb, Hd, Hm, Hmeg, Sc, Sg	Inundative	Registered	Yes	*
	Borers	Synanthedon spp. (10)	Fruit trees and ornamentals	Hb, Sc, Sf	Inundative	Registered	Yes	*
	Citrus root weevil	*Pachnaeusspp.* (6)	Citrus, ornamentals	Sr, Hb	Inundative	Registered	Yes	*
	Corn rootworm	*Diabrotica* spp. (6)	Vegetables	Hb, Sc	Inundative	Registered	Yes	*
	Cranberry girdler	*Chrysoteuchia topiaria* (11)	Cranberries	Sc	Inundative	Registered	Yes	*
	Crane fly	Diptera: Tipulidae	Turf	Sc	Inundative	Registered	Yes	*
	Diaprepes root weevil	*Diaprepes abbreviatus* (6)	Citrus, ornamentals	Hb, Sr	Inundative	Registered	Yes	*
	Fungus gnats	Diptera: Sciaridae	Mushrooms, greenhouse	Sf, Hb	Inundative	Registered	Yes	*
	Grape root borer	*Vitacea polistiformis* (10)	Grapes	Hz, Hb	Inundative	Registered	No	*
	Iris borer	*Macronoctua onusta* (9)	Iris	Hb, Sc	Inundative	Registered	Yes	*
	Mole crickets	*Scapteriscus* spp. (12)	Turf	Sc, Sr, Scap	Inundative	Registered	Yes	*
	Scarab grubs	Coleoptera: Scarabaeidae	Turf, ornamentals	Hb, Sc, Sg, Ss, Hz	Inundative	Registered	Yes	*
	Strawberry root weevil	*Otiorhynchus ovatus* (6)	Berries	Hm	Inundative	Registered	Yes	*
	Sugarbeet weevil	*Temnorhinus mendicus* (6)	Sugar beets	Hb, Sc	Inundative	Registered	No	*
	Sweetpotato weevil	*Cylas formicarius* (6)	Sweet potato	Hb, Sc, Sf	Inundative	Registered	Yes	*
	Wireworms	Coleoptera: Elateridae	Vegetables	Hb, Hm, Sc	Inundative	Registered	Yes	[64]
**Arthropods**								
Carabid	Black vine weevil	*Otiorhynchus sulcatus* (6)	Strawberry	*Carabus nemoralis*	Conservation	Experimental	No	[65]
	Black vine weevil	*Otiorhynchus sulcatus* (6)	Strawberry	*Nebria brevicollis*	Conservation	Experimental	No	[65]
	Black vine weevil	*Otiorhynchus sulcatus* (6)	Strawberry	*Pterostichus algidu*	Conservation	Experimental	No	[65]
	Black vine weevil	*Otiorhynchus sulcatus* (6)	Strawberry	*Pterostichus melanarius*	Conservation	Experimental	No	[65]
	Black vine weevil	*Otiorhynchus sulcatus* (6)	Strawberry	*Scaphinotus marginatus*	Conservation	Experimental	No	[65]
	Western corn rootworm	*Diabrotica virgifera virgifera* (5)	Maize	*Pterostichus permundus*	Conservation	Experimental	Yes	[66]
	Western corn rootworm	*Diabrotica virgifera virgifera* (5)	Maize	*Poecilus chalcites*	Conservation	Experimental	No	[66]
	Western corn rootworm	*Diabrotica virgifera virgifera* (5)	Maize	*Cyclotrachelus alternans*	conservation	experimental	No	[66]
	Western corn rootworm	*Diabrotica virgifera virgifera* (5)	Maize	*Poecilus lucublandus*	conservation	experimental	No	[66]
Acari	Western corn rootworm	*Diabrotica virgifera virgifera* (5)	Maize	*Gaeolaelaps aculeifer*	conservation	experimental	No	[67]
Orthoptera	Western corn rootworm	*Diabrotica virgifera virgifera* (5)	Maize	*Allonemobius* spp.	conservation	experimental	No	[66]
Opiliones	Western corn rootworm	*Diabrotica virgifera virgifera* (5)	Maize	*Phalangium opilio*	conservation	experimental	No	[66]
Hymenoptera	Western corn rootworm	*Diabrotica virgifera virgifera* (5)	Maize	*Hymenoptera: Formicidae*	conservation	experimental	Yes	[66]
Hemiptera	Western corn rootworm	*Diabrotica virgifera virgifera* (*5*)	Maize	*Geocoris* sp.	conservation	experimental	No	[66]
Araneae	Western corn rootworm	*Diabrotica virgifera virgifera* (*5*)	Maize	Linyphiidae	conservation	experimental	No	[66]

^1^ 1 = Lepidoptera: Gelechiidae, 2 = Coleoptera: Scarabaeidae, 3 = Diptera: Tipulidae, 4 = Diptera: Sciaridae, 5 = Coleoptera: Chrysomelidae, 6 = Coleoptera: Curculionidae, 7 = Hemiptera: Phylloxeridae, 8 = Diptera: Anthomyiidae, 9 = Lepidoptera: Noctuidae, 10 = Lepidoptera: Sesiidae, 11 = Lepidoptera: Crambidae, 12 = Orthopetra: Gryllotalpidae; ^2^ Bt = *Bacillus thuringiensis*, Hb = *Heterorhabditis bacteriophora*, Hd = *H. downesi*, Hm = *H. marelatus*, Hmeg = *H. megidis* ,Hz = *H. zealandica*, Sc = *Steinernema carpocapsae*, Sf = *S. feltiae*, Sg = *S. glaseri*, Sk *= S. kushidai*, Sr = *S. riobrave*, Sscap = *S. scapterisci*, Ss = *S. scarabaei*, Mr = *Metarhizium robertsii*, Bb = *Beauveria bassiana*, If = *Isaria fumosorosea*, Mb = *Metarhizium brunneum*, Ma = *Metarhizium anispliae*. *: The list of EPN species used as biocontrol agents against root pests presented here was extracted from the exhaustive list presented in https://biocontrol.entomology.cornell.edu/pathogens/nematodes.php.
